# The Interaction Pattern of Murine Serum Ficolin-A with Microorganisms

**DOI:** 10.1371/journal.pone.0038196

**Published:** 2012-05-30

**Authors:** Tina Hummelshøj, Ying Jie Ma, Lea Munthe-Fog, Thomas Bjarnsholt, Claus Moser, Mikkel-Ole Skjoedt, Luigina Romani, Teizo Fujita, Yuichi Endo, Peter Garred

**Affiliations:** 1 Laboratory of Molecular Medicine, Department of Clinical Immunology, Rigshospitalet, Copenhagen, Denmark; 2 Department of Clinical Microbiology, Rigshospitalet, Copenhagen, Denmark; 3 Department of International Health, Immunology and Microbiology, Faculty of Health Sciences, University of Copenhagen, Copenhagen, Denmark; 4 Microbiology Section, Department of Experimental Medicine and Biochemical Science, University of Perugia, Perugia, Italy; 5 Department of Immunology, Fukushima Medical University School of Medicine, Fukushima, Japan; Institute of Medical Microbiology and Hospital Epidemiology-Hanover Medical School, Germany

## Abstract

The ficolins are soluble pattern recognition molecules in the lectin pathway of complement, but the spectrum and mode of interaction with pathogens are largely unknown. In this study, we investigated the binding properties of the murine serum ficolin-A towards a panel of different clinical relevant microorganisms (N = 45) and compared the binding profile with human serum ficolin-2 and ficolin-3. Ficolin-A was able to bind Gram-positive bacteria strains including *E. faecalis*, *L. monocytogenes* and some *S. aureus* strains, but not to the investigated *S. agalactiae (Group B streptococcus)* strains. Regarding Gram-negative bacteria ficolin-A was able to bind to some *E. coli* and *P. aeruginosa* strains, but not to the investigated *Salmonella* strains. Of particular interest ficolin-A bound strongly to the pathogenic *E. coli,* O157:H7 and O149 strains, but it did not bind to the non-pathogenic *E. coli,* ATCC 25922 strain. Additionally, ficolin-A was able to bind purified LPS from these pathogenic strains. Furthermore, ficolin-A bound to a clinical isolate of the fungus *A. fumigatus*. In general ficolin-2 showed similar selective binding spectrum towards pathogenic microorganisms as observed for ficolin-A indicating specific pathophysiological roles of these molecules in host defence. In contrast, ficolin-3 did not bind to any of the investigated microorganisms and the anti-microbial role of ficolin-3 still remains elusive.

## Introduction

The ficolins are recognition molecules in the lectin pathway of the complement system that bind to specific structures on microorganisms and self cellular debris enabling quick and simple aggregation and opsonization of hazardous material [1], [2].

The ficolins are highly oligomerized glycoproteins containing both collagen-like (CL) and fibrinogen-like (FBG) regions. As seen for mannose-binding lectin (MBL), the CL region is able to associate with the MBL/ficolin associated serine proteases (MASPs), which activate the lectin pathway of the complement system [1]. The ficolins are built up of several structural subunits each composed of three identical polypeptide chains [3]–[5]. Through the FBG domain they bind structures such as N-acetyl-glucosamine (GlcNAc), N-acetyl-galactosamine (GalNAc), and acetylated compounds located on the target cell [6], [7].

The ficolins have been described in both vertebrates and invertebrates where each species contain several paralogues ficolins (typical 2–4). In human, three ficolins are described: ficolin-1 (M-ficolin), ficolin-2 (L-ficolin), and ficolin-3 (H-ficolin). Ficolin-1 is present in low serum concentrations whereas ficolin-2 and ficolin-3 are present in concentrations of ∼5 µg/ml and ∼25 µg/ml, respectively [8]–[11]. Two ficolin molecules have been identified in mice termed ficolin-A and ficolin-B. Ficolin-B is expressed in myeloid bone marrow derived cells and ficolin-A is expressed in the liver and spleen and is present in serum [5], [12]. Based on the protein sequence and exon organisation it is likely that ficolin-B is the orthologue to the human ficolin-1, whereas both the mouse ficolin-A and the human ficolin-2 seems to have evolved independently from a common ficolin-1/ficolin-B lineage [13], [14]. The mouse homologue of the human ficolin-3 gene is identified as a pseudogene in mice and rats [15].

Both ficolin-1 and ficolin-2 have been shown to bind different bacteria strains such as *Staphylococcus aureus*, *Salmonella typhimurium*, *Streptococcus pneumonia*, *Streptococcus pyogenes* and *Streptococcus agalactiae* and are able to stimulate phagocytosis by polymorphonuclear neutrohpils and monocytes [16]–[18]. However, the anti-bacterial significance of ficolin-3 still remains to be determined.

In this study we investigated the binding of the murine serum ficolin-A towards a panel of different clinical relevant bacterial and fungal strains and compared this binding profile with that from human serum ficolin-2 and ficolin-3.

## Results

### Ficolin Quantification Assay

Since no commercial antibodies against ficolin-A are currently available for a quantification assay we optimized an ELISA setup based on acetylated bovine serum albumin (acBSA) as ligand molecule. Recombinant ficolin-A, ficolin-2 and ficolin-3 were able to bind acBSA in a dose dependent manner and this assay was used for determination of ficolin binding to microorganisms.

### Binding of Ficolins to Microorganisms

**Figure 1:**
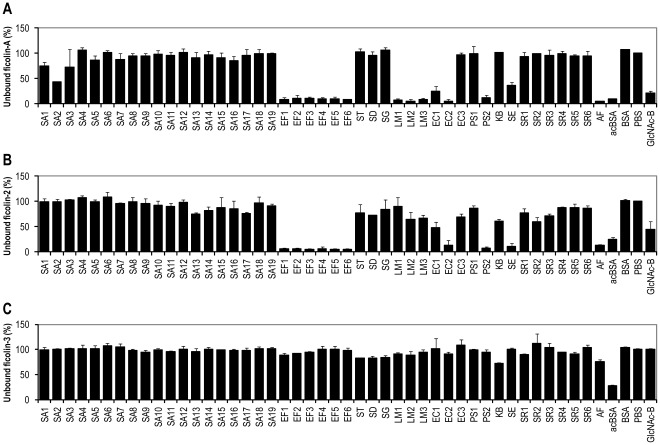
Binding of the ficolins to different microorganisms. After incubation of the microorganisms (1×10^8^ cells) with 0.2 µg of either recombinant ficolin-A (A), ficolin-2 (B) or ficolin-3 (C) the concentration of residual ficolin was estimated on acBSA coated polystyrene plates. Binding is indicated with decreased percentage signals related to PBS where 75–100% reflects no binding. Strong interaction between the ficolins and microorganisms is indicated with values below 10%. OD450nm values at 100% for the ficolin-A, ficolin-2 and ficolin-3 assays were 2.039, 0.920 and 1.641, respectively. Error bars indicate standard derivations of duplications. The experiment was repeated three times showing similar data.

The binding of mouse ficolin-A and human ficolin-2 and ficolin-3 were investigated towards a panel of different microorganisms (table 1). Binding was detected by incubating bacteria or fungus with ficolins and the residual ficolin protein in the supernatant after centrifugation was measured and the percentage of remaining ficolin was determined. A result of 100% indicates no binding to microorganisms whereas 0% indicates complete binding. Values between 100% and 75% were not considered as positive interaction between ficolins and microorganisms. Positive binding was determined to be percentage value below 75% and values below 10% were considered as very strong binding. Ficolin-A and ficolin-2 were able to bind several of the investigated microorganisms (Fig.1A and Fig.1B). Ficolin-A interacted with all the *Enterococcus faecalis* (EFs) strains and bound strongly to all the *Listeria monocytogenes* (LMs) strains (Fig.1A). It also binds to *Staphylococcus epidermidis* (SE) and *Aspergillus fumigatus* (AF). Ficolin-A selectively interacts with some strains of *Escherichia coli* (ECs) and *Pseudomonas aeruginosa* (PSs). Ficolin-2 interacted with all *E. faecalis* (EFs) strains as wells as the *S. epidermidis* (SE) and *A. fumigatus* (AF). As ficolin-A, ficolin-2 bound selectively to some *E. coli* (ECs) and *P. aeruginosa* (PSs) strains (Fig.1B) and to some degree to the *Salmonella* (ST, SD, SG) strains. At these settings, ficolin-3 did not interact with any of the bacteria strains investigated (Fig.1C). Control experiments demonstrated ficolin-3 binding to acBSA-coupled sepharose beads and not to BSA coupled sepharose beads.

**Table 1:** investigated microorganisms and ligands.

ABBAbbrev.	Name	Gram-classification	N
SA	*Staphylococcus aureus*	Positive	19
SR	*Streptococcus agalactiae * ***(*** *Group B* ***)***	Positive	6
EF	*Enterococcus faecalis*	Positive	6
LM	*Listeria monocytogenes*	Positive	3
SE	*Staphylococcus epidermidis*	Positive	1
ST	*Salmonella Typhimurium*	Negative	1
SD	*Salmonella Dublin*	Negative	1
SG	*Salmonella Gallinarium*	Negative	1
EC	*Escherichia coli*	Negative	3
PS	*Pseudomonas aeruginosa*	Negative	2
KB	*Klebsiella pneumoniae*	Negative	1
AF	*Aspergillus fumigatus*	Negative	1
acBSA-B	acetylated bovine serum albumin sepharose beads	–	–
BSA-B	bolive serum albumin sepharose beads	–	–
PBS	phosphate buffered saline	–	–
Mannose-B	mannose agarose beads	–	–
GlcNAc-B	N-acetyl-D-glycosamine agarose beads	–	–

**Figure 2:**
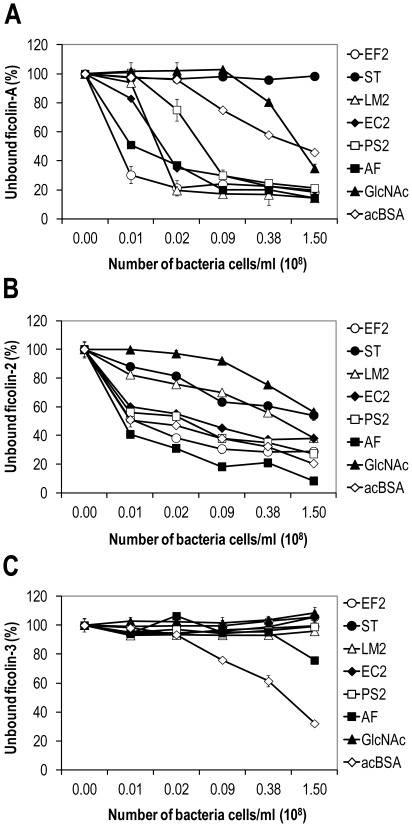
Dose-dependent binding of the ficolins (0.2 µg) to microorganisms. Ficolin-A showed strong binding to *E. faecalis* (EF2), *A. fumigatus* (AF), *L. monocytogenes* (LM1), *E. coli* (EC2) and *P. aeruginosa* (PS2) (A). Ficolin-2 showed binding to all six microorganisms (B). Ficolin-3 did not bind any of the bacteria but a weak binding was observed to *A. fumigatus* (AF) (C). GlcNAc-agarose beads and acBSA-coated sepharose beads were included as positive controls. Binding is indicated with decreased percentage signals related to PBS where 100% reflects no binding. OD450nm values at 100% for the ficolin-A, ficolin-2 and ficolin-3 assays were 1.263, 2.561 and 1.338, respectively. Error bars indicate standard derivations of duplicates. The results shown are typical of three experiments.

A serial dilution of the bacteria was incubated with fixed concentrations of the ficolins. A dose-dependent ficolin-A binding was confirmed to *E. faecalis* (EF2), *L. monocytogenes* (LM2), *E. coli* (EC2), *P. aeruginosa* (PS2) and *A.fumigutus* (AF) (Fig.2A). Binding of ficolin-2 was also confirmed to *E. faecalis* (EF2), *A. fumigatus* (AF), *E. coli* (EC2) and *P. aeruginosa* (PS2) although the dose-dependency was not as dynamic as seen for ficolin-A (Fig.2B). No binding to bacteria was detected for ficolin-3 (Fig.2C).

**Figure 3:**
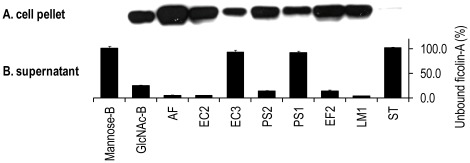
Binding of the ficolin-A to microorganisms. After incubation of the bacteria with recombinant ficolin-A the bacteria were washed three times and analysed on SDS-PAGE followed by western blotting (A). Binding was detected to GlcNAc-agaose beads (GlcNAc-B) but not mannose-agarose beads (Mannose-B). Strong binding was observed to *A. fumigatus* (AF), *E. coli* (EC2), *P. aeruginosa* (PS2), *E. faecalis* (EF2) and *L. monocytogenes* (LM1). Residual concentration of ficolin-A in the supernatant was analysed on acBSA-coated polystyrene plates (B). Binding is indicated by low percentage value where 100% reflects no binding. OD450nm value at 100% was 1.799. Error bars indicate standard derivations of two experiments.

Ficolin-A was incubated with relevant microorganisms and the bacteria cells were analysed on SDS-PAGE followed by western blotting (Fig.3A). Ficolin-A binding was confirmed to *E. faecalis* (EF2), *L. monocytogenes* (LM2), *E. coli* (EC2) and *P. aeruginosa* (PS2). Weaker binding was detected to *E. coli* (EC3) and *P. aeruginosa* (PS1). No binding was detected to *S. Typhimurium* (ST). Control experiments showed no binding to mannan-agarose beads but positive binding to GlcNAc-agarose beads. These data were supported by analyzing the supernatant containing the unbound protein on an acBSA matrix (Fig.3B).

### Binding of Serum Ficolin-A to Microorganisms

**Figure 4:**
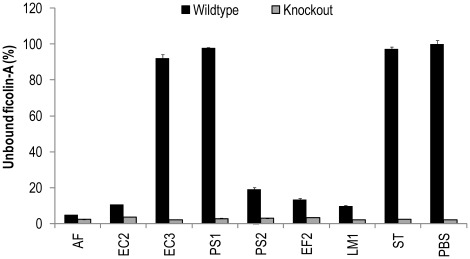
Binding of serum ficolin-A to microorganisms. Ficolin-A knock-out serum and wild type serum was incubated with different microorganisms. After incubation of the bacteria (1×10^8^ cells) with serum diluted 1∶50 in Barb-T buffer, the concentration of residual ficolin-A was estimated on acBSA coated polystyrene plates. Binding is indicated with decreased percentage signals where 100% reflects no binding. Binding was detected to *A. fumigatus* (AF), *E. coli* (EC2), *P. aeruginosa* (PS2), *E. faecalis* (EF2) and *L. monocytogenes* (LM1). OD450nm at 100% is 2.731. Error bars indicate standard deviations of duplicates.

Bacteria were incubated with ficolin-A knock-out serum or wildtype serum. Ficolin-A binding was detected in the wildtype serum to *A. fumigatus* (AF), *E. coli* (EC2), *P. aeruginosa* (PS2), *E. faecalis* (EF2) and *L. monocytogenes* (LM2) (Fig.4). These data confirm the results obtained with recombinant ficolin-A.

### Binding of Ficolin-A to LPS

**Figure 5:**
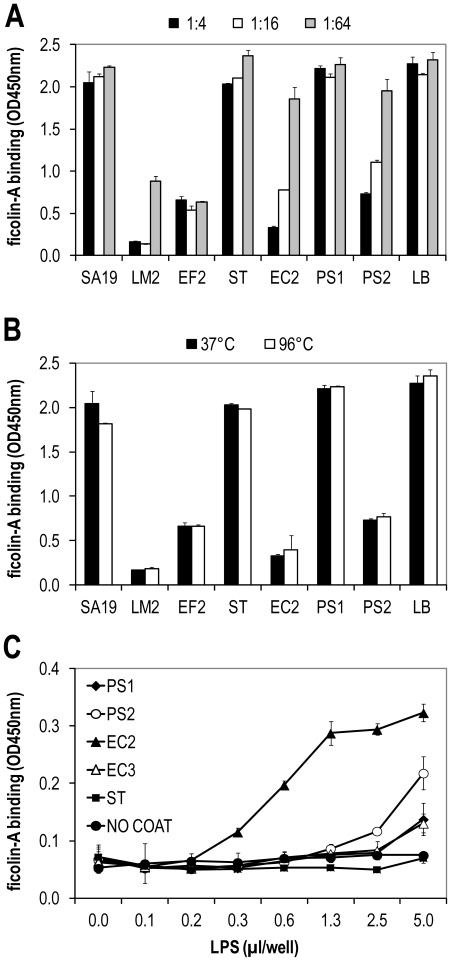
Binding of ficolin-A to bacterial components. The binding of ficolin-A (0.2 µg) to acBSA was inhibited with sterile filtrated overnight bacterial growth medium from some of the bacteria (A). No differences were observed when the growth medium was heat inactivated at 96°C (B). Purified LPS was coated on polystyrene plates and serum from ficolin-B knock-out serum diluted 1∶10 in Barb-T buffer was added (C). Ficolin-A binding to LPS from *E. coli* (EC2) was detected in a dose-dependent manner. Error bars indicate standard deviations of duplicates. Shown is one representative experiment of three.

Overnight bacteria culture was cleared from bacteria cells by centrifugation and the medium was sterile filtrated and incubated in different dilutions with ficolin-A and added to acBSA plates. The ficolin-A binding to the acBSA matrix was inhibited with increased concentration of bacteria growth medium from *L. monocytogenes* (LM2), *E. faecalis* (EF2), *E. coli* (EC2) and *P. aeruginosa* (PS2) (Fig.5A). However, no inhibition to acBSA was observed when incubating ficolin-A with growth medium from *S. aureus* (SA19), *S. Typhimurium* (ST) or *P. aeruginosa* (PS1). In order to investigate if the bacteria produce proteases that inactivate ficolin-A, and thereby could explain the observed reduced binding to acBSA, the bacteria overnight culture medium was heat inactivated, sterile filtrated and incubated with ficolin-A and measured on the acBSA plates (Fig.5B). No difference between the heat-inactivated growth medium and control growth medium was observed. These results indicate that the ficolin-A binding bacteria strains excrete substances such as LTA or LPS to the culture medium that inhibits the binding of ficolin-A to acBSA. LPS was purified from the investigated Gram-negative bacteria and coated on a polystyrene plate and ficolin-A was added in a serial dilution and the binding was detected. Ficolin-A bound LPS from *E. coli* (EC2) and to some degree to LPS from *P. aeruginosa* (PS2) (Fig5C).

### Binding of Ficolin-A to *A. Fumigatus* Using Flow Cytometry

**Figure 6:**
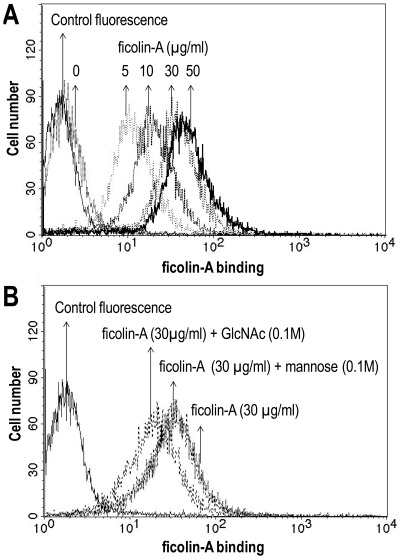
Binding of ficolin-A to *A. fumigatus* conidia using flow cytometry. *A. fumigatus* (1.4×10^6^ cells) were incubated with elevated concentration of ficolin-A (A). Bound protein was detected with polyclonal rabbit anti-mouse ficolin-A antibody. *A. fumigatus* (1.4×10^6^ cells) were incubated with ficolin-A (30 µg/ml) in the presence of GlcNAc or mannose, respectively (B). Bound ficolin-A was detected as depicted above. As a control, *A. fumigatus* was incubated in the absence of ficolin-A. Results are representative of three independent experiments that yield similar results.

Flow cytometry was used to examine the binding of ficolin-A to *A. fumigatus*. The microorganism was incubated with recombinant ficolin-A and detected with polyclonal rabbit anti-mouse ficolin-A (Japan) antibody followed by FITC-conjugated anti-rabbit IgG. Fig.6A shows a significant dose-dependent binding of ficolin-A to *A. fumigatus*. This binding was inhibited by GlnNAc but not mannose (Fig.6B).

### Binding of Ficolin-A to Microorganisms Using Confocal Laser Scanning Microscope (CLSM)

**Figure 7:**
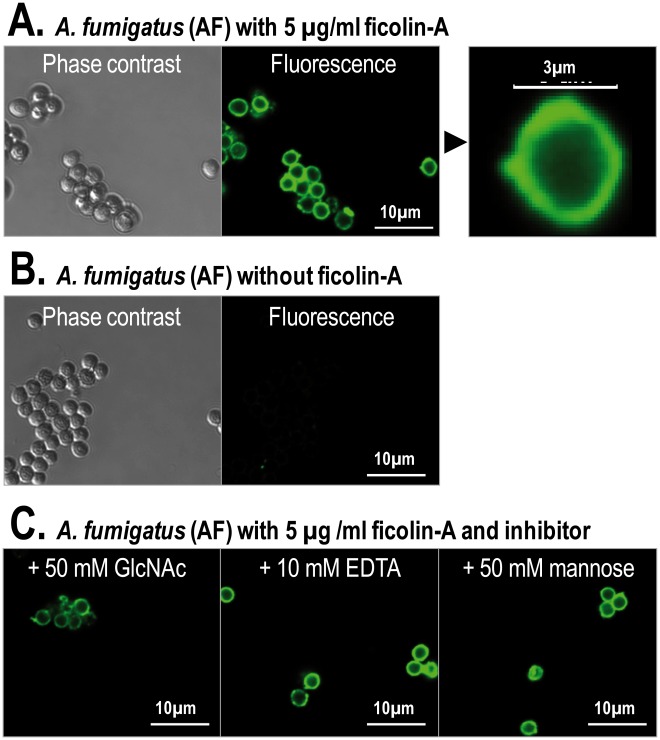
Binding of ficolin-A to *A. fumigatus* using confocal laser scanning fluorescence microscopy. *A. fumigatus* (4×10^6^ cells) was incubated with ficolin-A (5 µg/ml) (A) or without ficolin-A (B). The binding was inhibited with GlcNAc but not EDTA or mannose (C). The binding was detected with a monoclonal rat anti-mouse ficolin-A antibody.

[TIGHER]Interaction between ficolin-A and different microorganisms were assessed by CLSM. Ficolin-A bound strongly to all of the conidia cells of *A. fumigatus* (Fig.7A). No fluorescence was observed when incubating without ficolin-A (Fig.7B) or with ficolin-3 with respective antibodies (data not shown). The binding of ficolin-A to *A. fumigatus* was inhibited with GlcNAc but not EDTA or mannose (Fig.7C).

**Figure 8:**
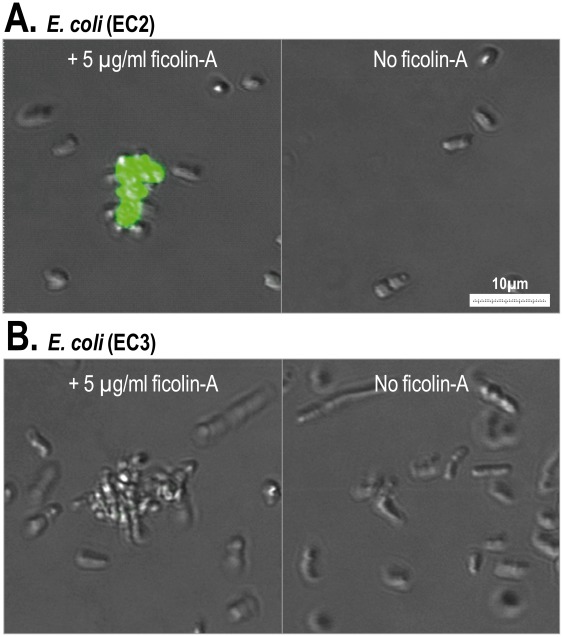
Binding of ficolin-A to *E. coli* using confocal laser scanning fluorescence microscopy. Ficolin-A (5 µg/ml) was incubated with *E. coli* EC2 (A) or *E. coli* EC3 (B). The bacteria (4×10^6^ cells) were incubated with or without ficolin-A. Ficolin-A binding was only observed to *E. coli* EC2 and this binding was found on aggregated bacteria. The binding was detected with a monoclonal rat anti-mouse ficolin-A antibody.

Ficolin-A binding was detected to *E. coli* (EC2), however this interaction was restricted to aggregated cells only (Fig.8A). All aggregated bacteria on the slide were positive. No binding of aggregated *E. coli* (EC3) was detected (Fig.8B). As expected based on the previous results, no binding was detected to *S. Typhimurium* (ST) (data not shown).

**Figure 9:**
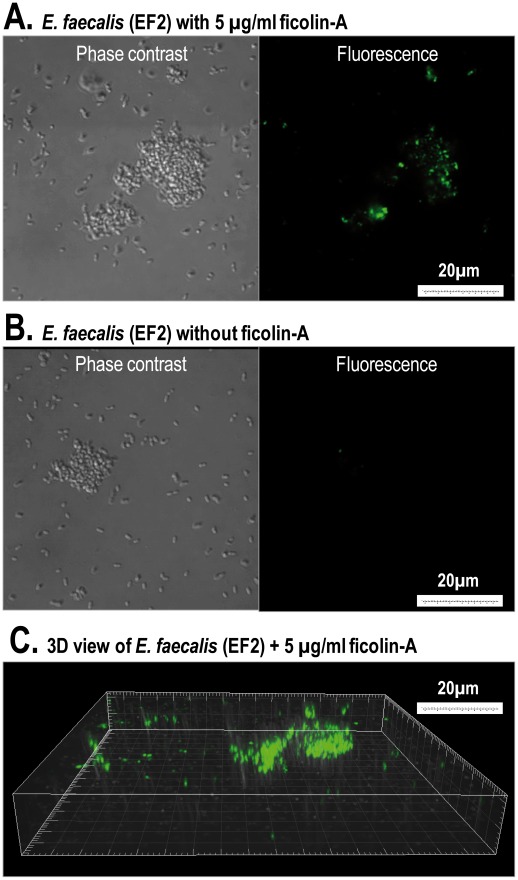
Binding of ficolin-A to *E. faecalis* (EF2) using confocal laser scanning fluorescence microscopy. The *E. faecalis* (4×10^6^ cells) was incubated with ficolin-A (5 µg/ml) (A) or without ficolin-A (B). Ficolin-A binding was only observed to aggregated *E. faecalis* cells. 3D view of the ficolin-A binding to aggregated bacteria (C). The binding was detected with a monoclonal rat anti-mouse ficolin-A antibody.

Ficolin-A also bound aggregates of *E. faecalis* (EF2) but not single cells (Fig.9A). No signal was detected when incubating without ficolin-A (Fig.9B). The binding appeared to be concentrated to large aggregates (Fig.9C).

## Discussion

The innate immune system consists of various types of cells and molecules that specifically interact with each other to initiate a quick and effective host defence. The murine ficolin-A and human ficolin-2 and ficolin-3 are serum proteins that bind to carbohydrate structures and acetylated compounds displayed on the surfaces of both mammalian tissues and on microorganisms [19]–[21]. Carbohydrates may be arranged into extremely variable structures, which on some microorganisms allow the ficolins to bind. The interactions between human ficolins and carbohydrates have been shown to be involved in activities such as opsonization, activation of complement and phagocytosis [2], [4]. In this study we investigated the binding of the mouse ficolin-A to a number of different clinical relevant microbial strains and compared this binding profile to human serum ficolin-2 and ficolin-3.

The results of this work demonstrate a broad binding profile of mouse ficolin-A towards both Gram-positive and Gram-negative bacterial strains. Significant ficolin-A binding was detected towards all the investigated *E. faecalis* and *L. monocytogenes* strains whereas only some of the *E. coli* strains and *P. aeruginosa* strains reacted with ficolin-A. Ficolin-A also showed a strong binding towards *S. epidermidis* and the fungus *A. fumigatus*. A similar binding profile was observed for human ficolin-2. However, this molecule also binds to *Salmonella* strains to some degree. In contrast, ficolin-2 did not bind *L. monocytogenes* in the same degree as ficolin-A. Ficolin-2 has previously been shown to bind to Gram-positive bacteria such as *S. pneumoniae*, *S. agalactiae* and *S. aureus*, and to Gram-negative bacteria such as *S. typhimurium* and *E. coli* [20], [22]. In this study we detected binding of ficolin-2 to only a few strains of *S.aureus* and the binding was apparently weaker to these strains compared to strains such as *E. faecalis* or some strains of *E. coli*. It has been shown that ficolin-2 only bind to capsulated *S. aureus* strains and not to noncapsulated strains [22]. Many bacteria, including both Gram-positive and Gram-negative, may be covered by an outer polysaccharide-containing layer termed the capsule layer. In the case of human pathogens, a large number of different capsule serotypes have been identified. For example, over 80 different capsular polysaccharides or K antigens have been described for *E. coli* [23], [24]. Various functions have been attributed to capsules including protection from desiccation [25] and adherence to surfaces and other bacteria contributing to biofilm formation [23]. Capsules often play a role in pathogenicity [26] acting as virulence factors to protect cells from phagocytosis and complement-mediated killing. The synthesis of the capsular material depends upon the environment and whether the bacteria cultures are able shift their morphology during growth. The bacteria used in this work were grown exponential overnight in LB medium and contains likely a mixture of both capsulated and noncapsulated strains. Investigating a *S. aureus* bacteria culture that has been optimized for production of capsules may show a stronger interaction as described by Krarup et al. [22]. The same issue may be relevant for other bacteria strains as the *Group B Streptococci* that previously has been shown to bind ficolin-2 [16], where we could only confirm a weaker binding to these strains. However, it may be difficult to compare “strength” of ficolin binding to bacteria with previous published papers since the assays have been performed with different setting. Previous reports have demonstrated ficolin-3 does not appear to have a broad binding specificity towards pathogens [27]. The only bacteria that have been shown to interact with ficolin-3 are *Aerococcus viridians* and *Hafnia alvei* [27], [28] and the anti-microbial binding profile of the human ficolins is thereby very different. Supporting these data, the carbohydrate binding specificity of the human ficolins has been analyzed by glycan array screening demonstrating that ficolin-2 bind several different carbohydrates such as disulfated N-acetyllactosamine and tri- and tetra-saccharides containing terminal galactose or GlcNAc whereas ficolin-3 shows a very poor lectin activity [29]. Recently a mutation in the *FCN3* gene causing ficolin-3 deficiency has been observed in a patient with recurrent severe infections of different aetiologies [10] and ficolin-3 has also been shown to be important in tissue homeostasis [2], [30]. Thus it is at present unresolved whether ficolin-3 deficiency is directly associated with increased risk of infections or may aggravate the consequences of tissue damage caused by infections.

The group of *E. faecalis* bacteria appears to be a specific target for both ficolin-A and ficolin-2. *E. faecalis* is a opportunistic Gram-positive cocci that normally inhabits the intestinal tract but it may also be responsible for surgical wound infections and urinary tract infections (for review see [31]). Ficolin-A and ficolin-2 also bind to different small-colony variants (SCVs) (EF4, EF5, EF6) in a similar manner as the normal *E. faecalis* counterpart (EF3). SCVs constitute slow-growing subpopulations of the bacteria with distinct phenotypes having more pathogenic characteristics. It might be speculated that mouse ficolin-A and human ficolin-2 have an important function in controlling this pathogen. Furthermore, the binding was only detected to large aggregates of this bacteria and whether the interaction is directly to the bacteria cell wall or to biofilm remains to be investigated.

The binding of the serum ficolins was investigated towards three different *E. coli* strains; O149 (EC1), O157:H7 (EC2) and ATCC25922 (EC3). Both ficolin-A and ficolin-2 bind to the pathogenic *E. coli* strains O149 and O157:H7 but not the non-pathogenic *E. coli* ATCC25922. Supporting these data, ficolin-2 has recently been shown to bind the *E. coli* O104:H4 that were responsible for the recent outbreak of food poisoning in Germany causing haemolytic-uremic syndrome (HUS) [32].

The bacterium O157:H7 has been implicated in several outbreaks of hemorrhagic colitis included fatalities caused by haemolytic uraemic syndrome [33], [34] and the *E. coli* O149 is a pathogen that may lead to porcine postweaning diarrhea causing major problems in the pig industry worldwide [35]. The binding of ficolin-A to *E. coli* O157:H7 (EC2) was only detected to aggregated cells and not to single cells. *E. coli* strains are able to produce biofilm and the binding mechanism may be the same as observed for *E. faecalis*. Sterile filtrated overnight growth medium from the bacteria that associate with ficolin-A was able to inhibit the binding of ficolin-A to acBSA in a similar pattern as observed when using living bacteria. Therefore, we speculated whether the bacteria were able to produce enzymes that could inactivate the ficolin molecules. However, 96°C heat-inactivated bacteria growth medium showed a similar binding profile as observed with unheated growth medium. This strongly suggests that the reduced ficolin-A binding to acBSA was not due to a bacterial proteolytic digestion of ficolin-A but rather an affinity inhibition of the detection assay by secreted structures such as LPS or LTA. Ficolin-2 has been shown to bind LTA from *S. aureus*, *S. pyogenes* and *S. agalactieae* and thereby initiate the lectin pathway-dependent C4 turnover [20]. Furthermore, porcine ficolin-α react with LPS from different bacteria such as *E. coli*, *S. typhimurium*, *S. enteritidis* and *P.* aeruginosa and LTA from the *S. pyogenes* and *S. aureus* [18]. In order to study the binding of ficolin-A to endotoxins, we purified LPS from the investigated Gram-negative bacteria and measured the binding of LPS to ficolin-A. Ficolin-A was able to bind to LPS from the pathogenic *E. coli* O157:H7 (EC2) but not the non-pathogenic *E. coli* ATCC25922 (EC3). It might be speculated that LPS is the ligand structure for ficolin-A upon immune recognition of the pathogen. However, the binding of ficolins to released LPS from the pathogenic *E. coli* O157:H7 (EC2) could also be an evasion mechanism of the bacteria to hamper recognition and sequestration by innate immunity mechanisms as complement deposition, opsonization and phagocytosis.

We demonstrated a clear binding of ficolin-A towards *A. fumigatus.* We have previously shown that ficolin-2 binds to certain *A. fumigatus* strains [36] and this interaction was confirmed with new isolates in the present study. The binding of ficolin-A to *A. fumigatus* seems to be located to the cell membrane of the conidia and the binding was inhibited by GlcNAc but not mannose demonstrating that the interaction is through the FBG domain. These data may indicate that the contact between ficolin-A and the fungus is a result of a direct immune mechanism for tagging the microorganism for phagocytosis and complement deposition. The long pentraxin PTX3 has been shown to bind to *A. fumigatus* and to be critical against infection in mice models [37], [38]. We have previously demonstrated that ficolin-2 interacts with PTX3 [36] and that these two proteins might work synergistically against *A. fumigatus in vitro*. *A. fumigatus* has become one of the most prevalent airborne fungal pathogens, causing severe and usually fatal invasive infections in immunocompromised patients [39].

Even though ficolin-A and ficolin-2 are not considered as orthologues molecules based on evolutionary neighbouring three analyses [13], [14], the present results show that these two molecules might have similar properties. This suggest that ficolin-A knock-out mice models might be highly relevant for human disease model in general. However, one unresolved question that remains to be addressed is the possible role of the ficolins in viral infections and viral pathophysiology.

In conclusion, our results demonstrate that ficolin-A have a broad binding profile towards both Gram-negative and Gram-positive strains as well fungi. The binding properties towards clinical relevant pathogens suggest that ficolin-A is an important contributors to innate host defence against pathogens, but our findings also indicate that the microbes may be able to utilize the ficolins as virulence factors. Ficolin-A appears to share several of the ficolin-2 binding properties although some differences in the binding profile were observed. Ficolin-3 however, seems to have a very restricted anti-microbial profile.

## Materials and Methods

Maxisorp polystyrene microtiter plates were from NUNC (Roskilde, Denmark). PBS-buffer (10 mM Na_2_HPO_4_, 1.47 mM KH_2_PO_4_, 137 mM NaCl, 2.7 mM KCl, pH = 7.4) and Barbital-buffer (4 mM C_8_H_11_N_2_NaO_3_, 145 mM NaCl, 2.6 mM CaCl_2_, 2.1 mM MgCl_2_, pH = 7.4) were from the hospital pharmacy (RegionH Apoteket, Rigshospitalet, Copenhagen, Denmark). Tween-20 was from Merck (Hohenbrunn, Germany). FITC-conjugated swine anti-rabbit IgG was purchased from DAKO (Glostrup, Denmark). Goat anti-rat IgG Alexa Fluor 488, NuPAGE 3–8% Tris-acetate gels, Tris-acetate buffer, LDS loading buffer and reducing agent were purchased from Invitrogen (Taastrup, Denmark). HRP-conjugated streptavidin, HRP-coupled anti-rabbit immunoglobulin, nitrocellulose membranes, ECL hyperfilm and CNBr activated sepharose beads were from GE Healthcare (Buckinghamshire, United Kingdom). SuperSignal West Femto Maximum Sensitivity Substrate and Ready-to-use TMB substrate were from Pierce Biotechnology (Rockford, IL, USA). Chloramphenicol, tryptone, yeast extract, mannose agarose beads, GlcNAc agarose beads and GlcNAc were from Sigma (Brondby, Denmark). LPS Extraction Kit was from CHEMBIO (Hertfordshire, UK). Sterile filter 0.2 µm was from VWR (Herlev, Denmark). Sterile 40 µM cell strainer and the BD FACSCalibur were from Becton Dickinson (Bilthoven, The Netherlands). The monoclonal antibodies were produced in house; rat anti-mouse ficolin-A (FCNA-K12), mouse anti-human ficolin-2 (FCN219), mouse anti-human ficolin-3 (FCN334)). Polyclonal rabbit anti-mouse ficolin-A (Japan) antibody was produced in house. In selected experiments sera from ficolin-A and ficolin-B knock-out mice were used (the construction and characterisation of these mice will be reported elsewhere).

### Recombinant Ficolins

Recombinant mouse ficolin-A was synthesized as previously described for ficolin-2 and ficolin-3 [3], [40]. Briefly, the *FCNA* gene was amplified from mouse liver cDNA using the forward primer 5′-ctagctcgacgcgagatgcagtggcctacgctgt-3′ and the reverse primers 5′-tccggaattccgttaagatgctcggattttcatctc-3′. The constructs were cloned into the expression vector pED.dC and transfected into the Chinese hamster ovary (CHO) cell line CHO-DG44 (a kind gift from professor Lawrence Chasin, Columbia University, New York) using dhfr selection system. Subsequently, the recombinant ficolin-A were purified from the cell supernatant on GlcNAc agarose beads. Culture supernatants were centrifuged for 20 min at 15,000 g to obtain cleared supernatants and then loaded onto a column containing GlcNAc-agarose beads, which was been equilibrated with Barb-T buffer (Barbital-buffer with 0.05% Tween-20). The beads were washed with 100 times column volume of Barb-T buffer and the ficolins were eluted with 0.25 M GlcNAc in PBS buffer and dialyzed first into PBS containing 5 mM EDTA buffer and then pure PBS.

### Coupling of acBSA and BSA to Sepharose Beads

A total of 0.1 g dry CNBr-activated Sepharose 4B beads were allowed to swell in 1 mM HCl. After washing in 1 mM HCl, the beads were incubated with 4 mg BSA or acetylated BSA for 4 h at 4°C in a buffer composed of 0.1 M NaHCO_3_ and 0.5 M NaCl (pH 8.3). The beads were washed three times with the same buffer and blocked with 1 M ethanolamine. The beads were washed in 0.1 M TRIS-HCl, 0.5 M NaCl (pH 8.5).

### Bacteria

The following bacteria strains were examined in the assays: *Staphylococcus aureus* (SA1–19∶ 8325-4, SH1000, COL, EMRSA, SA564, 15981, USA300, Na34, m70, m124, m332, m137, Mu50, M9, ST398, S84F9, UAMS-1, P1, ATCC25923), *Escherichia coli* (EC1-3: O149, O157:H7, ATCC25922), *Enterococcus faecalis* (EF 1-6: TX0052, OG1RF, HEF5, HEF5-scv1, HEF5-scv2, HEF5-scv3), *Listeria monocytogenes* (LM1-3∶10403S, ScottA, LO28), *Salmonella Typhimurium* (ST1∶4/74), *Salmonella dublin* (ST2∶3246), *Salmonella gallinarium* (ST3: G9), *Pseudomonas aeruginosa* (PS1-2: ATCC27853, 53381), *Streptococcus agalactiae* (Group B streptococcus) (SR1-6∶33304, 33327, 33354, 54381, 33999, 33061), *Staphylococcus epidermidis* (clinical isolate A33), and *Klebsiella pneumoniae* (KB1∶51046). All strains were grown in standard LB (Luria-Bertani broth) medium.

### Preparation of *A. Fumigatus* Conidia

The *Aspergillus fumigatus* strain was obtained from a clinical isolate 6871 (University of Perugia, Italia). The *A. fumigatus* was grown on Sabouraud dextrose agar supplemented with chloramphenicol by agar-streak for 4 days at 28°C. Abundant conidia were obtained under these conditions. The conidia were harvested by washing the slant culture with PBS containing 0.05% Tween-20 (PBS-T) and gently scraping the conidia from the mycelium with sterile cotton-tipped applicators. The conidia were then allowed to settle by gravity followed by filtration through sterile 40 µM cell strainer to remove hyphal fragments and cell debris. After extensive washing with PBS-T, the conidia were counted and diluted to the desired concentrations. The *A. fumigatus* was heat-inactivated at 121°C for 15 min prior to use.

### Consumption Assay

Bacteria were incubated overnight at 37°C, shaking at 130 rmp in 5 ml LB-medium. The overnight cultures were centrifuged at 9,000 g for 5 min and washed three times in PBS. The cell pellet was resuspended in Barb-T buffer and the bacteria concentration was estimated spectrophotometric at OD600nm, where absorbance value 1 corresponds to approximately 1×10^9^ cells/ml. Bacteria (1×10^8^ cells) or fungus (1×10^7^ cells) were incubated shaking for 2 h at 37°C with 0.4 µg/ml rficolin-A, rficolin-2 or rficolin-3 in Barb-T buffer in a total volume of 500 µl. After centrifugation (9,000 g, 5 min) the supernatant was transferred to ficolin-A, ficolin-2 and ficolin-3 quantification assays described below. The percentage of unbound ficolin was estimated by dividing the signal from the sample containing the microorganisms with the signal from the control sample without microorganisms. Each test was repeated three times in duplicates. In some experiments, ficolin-A wildtype or ficolin-A knock-out mice sera (1∶50) were incubated with bacteria and analysed as described above. The bacteria pellet was washed three times in Barb-T buffer and resuspended in SDS-PAGE loading buffer and analyzed by western blotting.

### Ficolin-A, Ficolin-2 and Ficolin-3 Quantification Assay

Microtiter plates were coated with 100 µl of 5 µg/ml acBSA in PBS-buffer overnight at 4°C. Plates were blocked for 1 h with Barb-T and subsequently washed three times in Barb-T. Samples were added to the wells and incubated for 2 h at 37°C. Ficolin-A was detected with 0.25 µg/ml polyclonal anti-mouse ficolin-A (Japan), ficolin-2 was detected with 0.5 µg/ml biotinylated monoclonal FCN219 and ficolin-3 was detected with 0.25 µg/ml biotinylated monoclonal FCN334. Plates were incubated for 1 h at 37°C. After washing, HRP-coupled anti-rabbit immunoglobulin (0.25 µg/ml) or HRP-conjugated streptavidin (1∶6000) were added for 1 h at 37°C. Plates were washed and developed for 10–15 min with TMB substrate and stopped with 50 µl sulphuric acid/well. The optical density was measured at 450 nm.

### Dose Dependent Binding of Ficolins to Bacteria

Serial dilutions of overnight bacteria cultures (4-fold dilutions starting from 1.5×10^8^ cells) were incubated for 2 h at 37°C, shaking with 0.4 µg/ml rficolin-A, rficolin-2 or rficolin-3 in Barb-T buffer in a total volume of 500 µl. After centrifugation (9,000 g, 5 min) the supernatants were transferred to ficolin-A, ficolin-2 and ficolin-3 quantification assays described above.

### Western Blotting

Bacteria pellet samples were diluted in SDS-buffer with reducing agent and heated for 5 min at 90°C and applied to SDS-PAGE using 3–8% Tris-acetate gels. The separated proteins were subsequently blotted onto nitrocellulose membranes and the membranes were blocked using a 5% skim milk solution in PBS-T for 1 h at room temperature shaking. After a brief wash in PBS-T, the membranes were incubated shaking for 1 h at room temperature with 0.25 µg/ml polyclonal rabbit anti-mouse ficolin-A (Japan) antibody. Following incubation, the membranes were washed thoroughly in PBS-T and subsequently incubated with 0.25 µg/ml anti-rabbit-HRP. Membranes were developed on ECL hyperfilm with SuperSignal West Femto Maximum Sensitivity Substrate.

### Sterile Filtration and Heat Inactivation of Bacteria Growth Medium

Overnight bacteria culture was centrifuged at 9,000 g for 5 min and the growth medium were sterile filtrated using 0.2 µm filters and heat inactivated at 96°C for 30 min.

### LPS Purification

LPS was purified from 5 ml of overnight bacteria cultures (*S. typhimurium*, *P. aeruginosa* and *E.coli*) using LPS Extraction Kit at standard conditions. LPS was dissolved in 50 µl PBS.

### LPS Assay

Purified LPS from *S. typhimurium*, *P. aeruginosa* and *E.coli* were coated in polystyrene plates in 2-fold dilutions starting from 5 µl/well. Plates were blocked for 1 h with Barb-T and were subsequently washed twice in Barb-T. Ficolin-B knock-out serum diluted 1∶10 in Barb-T buffer was added to the wells and incubated for 2 h at 37°C. Ficolin-A was detected with 0.25 µg/ml polyclonal rabbit anti-mouse ficolin-A (Japan) antibody for 1 h at 37°C. After washing, HRP-coupled anti-rabbit immunoglobulin (0.25 µg/ml) was added for 1 h at 37°C. Plates were washed and developed for 10–15 min with TMB substrate. Addition of 50 µl/well sulphuric acid terminated the enzymatic reaction. The optical density was measured at 450 nm.

### Flow Cytometry

*A. fumigatus* conidia (1.4×10^6^ cells) were washed and resuspended in TBS-Mg^2+^ (10 mM Tris/150 mM NaCl/1.5 mM CaCl_2_/1 mM MgCl_2_, pH 7.4) buffer containing 1% heat-inactivated FCS (TBS-Mg^2+^/HI-FCS), followed by incubation with elevated concentration of mouse ficolin-A at 37°C for 1 h. All reaction volumes were 100 µl and *A. fumigatus* were washed after each step in cold TBS-Mg^2+^/HI-FCS. Bound proteins were detected with polyclonal rabbit anti-mouse ficolin-A (Japan) antibody by incubation at 4°C for 30 min, followed by 15 min of incubation at 4°C with FITC-conjugated anti-rabbit IgG. Finally, the *A. fumigatus* were washed and resuspended in 200 µl of cold TBS-Mg^2+^/HI-FCS. Flow cytometry was performed at the BD FACSCalibur and data was analyzed by BD CellQuest Pro software. In some experiments, *A. fumigatus* (1.4×10^6^ cells) were incubated with mouse ficolin-A (30 µg/ml) in TBS-Mg^2+^/HI-FCS in the presence of GlcNAc (0.1 M) and mannose (0.1 M), respectively. Bound ficolin-A was detected as depicted above. As a control *A. fumigatus* was incubated in the absence of mouse ficolin-A.

### Confocal Laser Scanning Fluorescence Microscopy

Ficolin-A (5 µg/ml) was incubated with 4×10^6^ microbial cells in Barb-T buffer in a total volume of 200 µl for 1 h at 37°C. After centrifugation (9,000 g, 5 min) the cell pellet was incubated with an in house produced monoclonal rat anti-mouse ficolin-A antibody (FCNA-K12) for 1 h at 37°C. After centrifugation (9,000 g, 5 min) the cell pellet was washed and incubated with anti-rat IgG Alexa Fluor 488 for 1 h at 37°C. The cells were washed and the samples were examined using a Zeiss LSM 710 confocal laser scanning microscope (CLSM) equipped with an Argon laser for excitation of the Alexa Fluor 488. The images were further processed using the IMARIS software package (Bitplane AG).

### Ethics Statement

This study was conducted in accordance with Danish law for animal protection and with the European Communities Council Directive 86/609/EEC for the protection of animals used for experimental purposes. The protocol was approved and experiments were permitted by the local government, The Animal Experiments Inspectorate, The National Authority, Denmark (permission No. 2009/561-1616) to be executed at the Department of Experimental Medicine, Faculty of Health Sciences, University of Copenhagen.
